# Awareness and mitigation measures on highly pathogenic avian influenza in pastoral poultry flocks of North‐central Nigeria: any challenging gap?

**DOI:** 10.1002/vms3.67

**Published:** 2017-06-30

**Authors:** Nma B. Alhaji, Suleiman Yatswako

**Affiliations:** ^1^ Department of Veterinary Public Health and Preventive Medicine University of Ibadan Ibadan Nigeria; ^2^ Niger State Avian Influenza Control Project Desk Office Veterinary Hospital Complex Minna Nigeria

**Keywords:** Biosecurity measures, HPAI H5N1, Epidemiology, Pastoralists, poultry flock, Nigeria

## Abstract

Village poultry closely interact with wild birds and other livestock in extensively managed poultry flocks, a practice common in pastoral communities of Nigeria. This practice provides sustained dissemination of avian viruses, such as highly pathogenic avian influenza (HPAI) H5N1 virus. The objectives of this study were to assess their knowledge/awareness, risks identification and biosecurity measures on HPAI H5N1 in pastoral poultry flocks. A questionnaire‐based cross‐sectional survey was conducted in systematically selected pastoral households of North‐central Nigeria between May 2015 and June 2016. A total of 422 pastoralists participated in the study. Mean age of the respondents was 54.7 ± 11.4 SD years and 36.0% of them were in age group 50–59 years. The majority (81.3%) of respondents were of the Fulani tribe. Also, 64.9% of the respondents had no formal education and only 6.9% had tertiary education. About 30.8% of the nomadic and 81.0% of sedentary pastoralists significantly mentioned avian influenza to be a zoonotic disease. Very few nomadic (10.9%) and sedentary (26.1%) pastoralists significantly reported restriction of birds’ movement to nearby water bodies as biosecurity measure. Only 7.6% of the nomadic and 16.1% of sedentary pastoralists practiced keeping of birds according to species. Sedentary pastoralists were more likely to have significant knowledge about HPAI H5N1 than the nomadic (OR: 1.76; 94% CI: 1.19–2.61). Female pastoralists were more likely to practice significant biosecurity measures against HPAI H5N1 than the males (OR: 1.99; 95% CI: 1.28–3.09). The majority of pastoralists neither possessed adequate knowledge about avian influenza nor applied adequate biosecurity measures against it, which are the most challenging gaps. Education of pastoralists on HPAI virus infection, specifically on information about clinical signs of avian influenza in birds, transmission dynamics among different species of birds, flyways of migrating wild birds and adequate mitigation measures are recommended.

## Introduction

The poultry population in Nigeria is estimated at 165 million, with backyard or ‘village’ poultry population, including those kept by pastoralists, constituting 84% (Federal Livestock Department (FLD) 2010; Food and Agriculture Organization of the United Nations (FAO) 2015). Previous epidemics of highly pathogenic avian influenza (HPAI) H5N1 that occurred in Nigeria between 2006 and 2008 affected 25/37 of the states in the country, with the destruction of millions of poultry and approximately 5.4 million USD paid in compensation by the Government of Nigeria (FAO 2015). Following a recent global wave of HPAI H5N1 spreading to newly affected countries, OIE confirmed the re‐emergence of H5N1 HPAI in Nigeria in January 2015. In the present outbreak, the disease has spread to nearly 400 enterprises and farms in 26 of the 37 states in the country and probably to bordering countries, such as Cameroon, Chad, Niger and Benin Republic (FAO 2015, 2016). This event is particularly critical given the importance of the Nigerian poultry industry to livelihood and food security.

The H5 of the current virus strain in Nigeria was identified as belonging to Clade 2.3.2.1c, which has been reported from several Asian countries in recent years. This clade has been associated in early 2015 with H5N1 cases in wild birds and poultry reported from Bulgaria and wild birds in Romania (Monne *et al*. 2015). The source of incursion of the H5N1 virus into Nigeria is difficult to determine and may have been related to the informal poultry trade in Nigeria or migratory bird movements (FAO 2015).

Village or backyard poultry closely interact with wild birds and other livestock, where in some settings, there is evidence of sustained dissemination of avian viruses, such as the HPAI, among extensively managed poultry flocks (Loth *et al*. 2011). Extensive management system is a common practice among pastoral communities of Nigeria, and disease surveillance in such communities is usually difficult because human populations are relatively small and highly mobile, moving with their livestock across large areas with few roads and modern means of communications (de Leeuw *et al*. 1995).

Scientifically based information on pastoralists’ knowledge/awareness about biosecurity and its practice, particularly against HPAI H5N1 in pastoral scavenging poultry flocks in Nigeria is not readily available. Yet, free‐range production is very important in the HAPI H5N1 transmission link between domestic and wild birds (Gilbert *et al*. 2006). Control and prevention of infectious diseases depend heavily on peoples’ compliance with recommendations on precautionary behaviour. This depends on the level of perceived risk and peoples’ understanding and willingness to adopt precautionary measures (Leppin & Aro 2009). Adequate poultry keepers’ knowledge on factors predisposing their birds to the risk of HPAI H5N1 virus infection can assist in the development of surveillance and interventions which will mitigate the virus transmission among backyard poultry flocks and humans in developing countries (Ismail & Ahmed 2010).

This study was aimed at assessing pastoralists’ knowledge/awareness, risk identification and biosecurity measures practice for HPAI H5N1 in potentially exposed pastoral poultry flocks in North‐central Nigeria. Our null hypothesis was that socio‐demographic characteristics of pastoralists do not influence their overall knowledge/awareness and biosecurity measures practised towards HPAI H5N1 in pastoral poultry flocks. We also hypothesized that the sedentary pastoralists do not possess more knowledge of or practised adequate mitigation measures for HPAI H5N1 than the nomadics. The findings of this study were expected to provide background information on knowledge base and mitigation practices for HPAI H5N1 in pastoral communities, which would serve as guide in designing surveillance programmes and biosecurity strategies against the disease in hard‐to‐reach rural settlements in developing countries.

## Materials and methods

### Study area and populations

**Figure 1:**
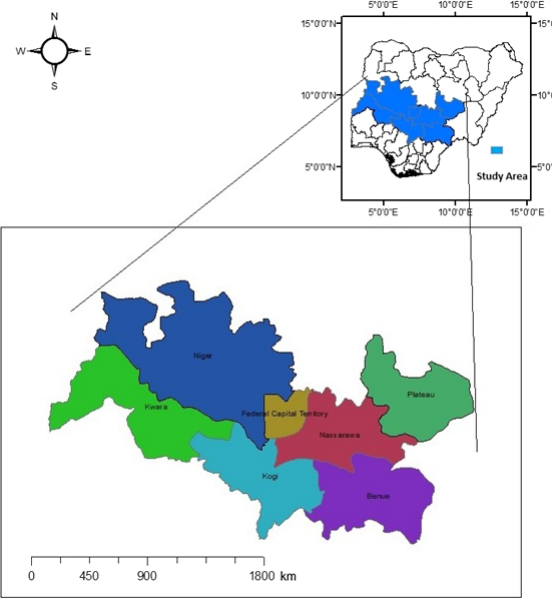
Map of North‐central zone of Nigeria (study area)

The study was conducted in the North‐central geo‐political zone of Nigeria. The zone consisted of seven states, namely Benue, Niger, Kwara, Kogi, Nasarawa and Plateau States as well as Abuja (the Federal Capital) (Fig. 1). Target populations were both the nomadic and sedentary pastoral households which kept poultry flocks in their communities and were domiciled in the zone during survey period. Each community had a minimum of 28 households deriving their livelihoods mainly from herding cattle, but kept local poultry at subsistence levels and cultivated few food crops. Study eligibility was based on a participant being a household head or spouse with minimum of 10 birds in a flock. Participants had to be 30 years of age or above. They were expected at these ages to possess existing veterinary knowledge and traditional oral history on livestock health and production management (Mariner & Paskin 2000).

For the purpose of this research, a nomadic pastoral household was defined as a household that kept mainly cattle, usually a large herd of 50 cattle and above, at least 10 local birds and took part in year‐round long movements over large ranges for grazing without a permanent homestead. A sedentary (agro‐pastoral) pastoral household was defined as a semi‐settled household with less than 50 cattle in herd, cultivating few crops, but keeping at least 15 local birds and having limited movements on low‐range grazing within their environments.

### Study design, sample size and sampling procedure

A questionnaire‐based cross‐sectional survey was conducted in randomly selected pastoral households between May 2015 and June 2016. The sample size was calculated using OpenEpi 2.3 software (Dean *et al*. 2009), with power set at 50% and 5% margin of error at 95% confidence level. A sample size of 384 households was obtained. A 10% contingency was added to take care of non‐response, and 422 households were enrolled into the study. Thirty pastoral communities were purposely selected across the study area, with at least four from each of the six States and Abuja. Systematic random sampling method was used to select the households from the communities. Sampling interval of two was used, obtained by dividing the total number of expected households (*n* = 28) in each community by the desired number of households to be sampled in it (*n* = 14).

### Questionnaire design and data collection

A structured questionnaire containing 37 questions, mostly close‐ended, to ease data processing, minimize variation and improve precision of responses was designed (Thrusfield 2009). It contained questions that elicited the following details: pastoralist's socio‐demographic characteristics (age, gender, marital status, occupation and formal education); knowledge/awareness about HPAI H5N1, its clinical signs, zoonotic nature and routes of transmission among birds; identification of risk factors that predisposed to HPAI H5N1 virus infection in poultry flocks; and biosecurity measures practised. The questionnaire was designed in English and verbally translated into local *Hausa* language during the process of questioning, as most of pastoralists did not possess formal education. Six trained teams of two enumerators were recruited and carried out interviewer‐administered questionnaires, with each interview lasting approximately 30 min and completed in each community on a single visit.

Respondents were provided with verbal information on the objectives of the study. Their informed consent was obtained verbally before commencement of each section of questionnaire administration and none declined to participate in the study. They were assured of voluntary participation, confidentiality of responses and the opportunity to withdraw at any time without prejudice in line with the Helsinki Declaration (World Medical Association Declaration of Helsinki (WMADH) 2001). Advocacy visits were made to each community a week prior to the proposed interview and necessary permission obtained from Ardos (community leaders). The study protocols were approved by the Niger State Ministry of Livestock and Fisheries Development Research Ethics Committee.

### Data management and analysis

Participants’ responses were first summarized into Microsoft Excel 7 (Microsoft Corporation, Redmond, WA, USA) spreadsheets. Data were analysed using EpiInfo 3.4.3 (CDC, Atlanta, GA, USA) and the Open Source Epidemiologic Statistics for Public Health (OpenEpi) version 2.3 (Dean *et al*. 2009). Frequencies and proportions were used for descriptive analysis. Categorical response variables from the pastoralists were presented as proportions and their associations determined by Chi‐square tests.

Independent (explanatory) variables were created from socio‐demographic characteristics of the participants in the questionnaires; while the response levels for overall knowledge/awareness and biosecurity measures practised assessed constituted the dependent (outcome) variables. However, to create outcome variables, a unique scoring system was used for the responses. Each respondent was assigned a response score within a range of 1–20 points and converted to 100%. These scores reflected stringency of their responses to questions. The score range was further categorized into ‘poor’ or ‘satisfactory’ to keep them as binary variables. Response scores that fell within 1–10 points were considered ‘poor’ (≤49%), and those that fell within 11–20 points were considered ‘satisfactory’ (≥50%).

Associations between the explanatory and outcome variables were first subjected to univariate analysis using Chi‐square tests (Dohoo *et al*. 2009). Factors found to be statistically significant at univariate analysis were finally subjected to likelihood stepwise backward multivariable logistic regression models to control for confounding and test for effect modification. The Hosmer and Lemeshow test was used to assess for goodness of fit of the final model and was found to be good. *P* < 0.05 was considered statistically significant in all analyses.

## Results

### Socio‐demographic characteristics of participants and poultry management

A total of 422 pastoralists participated in the study with a mean age of 54.7 ± 11.4 SD years, and most (36.0%) were in age group 50–59 years. Thirty one per cent (*n* = 131) of the respondents were males and 73.4% (*n* = 310) were married, while 6.2% (*n* = 26) and 20.4% (*n* = 86) were single and widows, respectively. Both nomadic and sedentary pastoralists participated equally (50%, *n* = 211 each). Most (81.3%, *n* = 343) of the participants were of the Fulani tribe. The majority (64.9%, *n* = 274) of the participants had no formal education and only very few (6.9%, *n* = 29) had tertiary education. Poultry keeping was reported by all respondents to have socio‐economic impacts on their livelihood.

**Figure 2:**
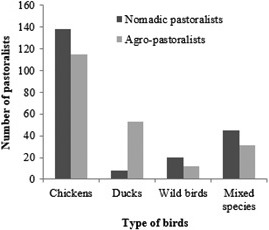
Occupational distribution of pastoralists interviewed, stratified by number of birds kept in pastoral communities of North‐central Nigeria: 2015–2016

Segregated by occupation, 65.4% (138/211) of the nomadic pastoralists kept chickens and 9.5% (20/211) kept wild birds like guinea fowls. Further, proportions of the sedentary pastoralists that kept chickens and mixed bird species were 54.5% (115/211) and 13.7% (31/211), respectively (Fig. 2).

### Pastoralists’ knowledge/awareness about HPAI H5N1

A significant majority (*P* < 0.05) of the nomadic (82.5%, 174/211) and sedentary (96.2%, 203/211) pastoralists reported to have heard about HPAI H5N1, which was locally called ‘*muran tsuntsuaye*’ in the study zone. Of all the pastoralists, only 24.2% (102/422) of them significantly mentioned to have heard about the disease from radio, while 20.9% (88/422) significantly heard from relations and 44.3% (187/422) from the veterinary health officials. Less than half (47.9%, 101/211) of the nomadics and over two‐thirds (86.3%, 182/211) of the sedentary pastoralists stated that HPAI H5N1 outbreaks had occurred in Nigeria. Very few of the nomadic (6.2%, 13/211) and sedentary (23.2%, 49/211) pastoralists indicated they had seen clinical signs of the disease. Additionally, 30.8% of nomadic and 81.0% of the sedentary pastoralists mentioned that avian influenza can be transmitted from birds to humans (zoonotic). More than two‐thirds of the sedentary pastoralists and less than one‐third of the nomadic pastoralists mentioned that the H5N1 can be associated with high morbidity and mortality in infected poultry flocks. However, about one‐third (33.6%, 71/211) of the nomadic and over two‐thirds (92.4%, 195/211) of sedentary pastoralists significantly reported that avian influenza can be controlled/prevented in pastoral bird flocks (Table 1).

**Table 1:** Pastoralists’ knowledge/awareness about avian influenza (H5N1) in pastoral communities of North‐central Nigeria: 2015–2016

Variable	Pastoralists	Yes *n* (row %)	No *n* (row %)	*P*‐value
Heard about avian influenza (H5N1) previously	Nomadic	174 (82.5)	37 (17.5)	0.001^a^
	Sedentary	203 (96.2)	8 (3.8)	
Avian influenza (H5N1) outbreaks have previously occurred in Nigeria	Nomadic	101 (47.9)	110 (52.1)	<0.001^a^
	Sedentary	182 (86.3)	29 (13.7)	
Have seen birds with clinical signs indicative of avian influenza	Nomadic	13 (6.2)	198 (93.8)	0.001^a^
	Sedentary	49 (23.2)	162 (76.8)	
Avian influenza can be transmitted from birds to humans (zoonosis)	Nomadic	65 (30.8)	146 (69.2)	<0.001^a^
	Sedentary	171 (81.0)	40 (19.0)	
Avian influenza is associated with high morbidity	Nomadic	52 (24.6)	159 (75.4)	<0.001^a^
	Sedentary	196 (92.9)	15 (7.1)	
Avian influenza is associated with high mortality	Nomadic	63 (29.9)	148 (70.1)	<0.001^a^
	Sedentary	184 (87.2)	27 (12.8)	
Avian influenza can be controlled or prevented	Nomadic	71 (33.6)	140 (66.4)	<0.001^a^
	Sedentary	195 (92.4)	16 (7.6)	
**Modes of transmission of H5N1 virus among birds**				
Contacts of healthy birds with blood of suspected ones	Nomadic	33 (15.6)	178 (84.4)	<0.001^a^
	Sedentary	157 (74.4)	54 (25.6)	
Contacts of healthy birds with faeces of suspected ones	Nomadic	18 (8.5)	193 (91.5)	<0.001^a^
	Sedentary	69 (32.2)	142 (67.3)	
Contacts of healthy birds with nasal discharges of suspected ones	Nomadic	10 (4.7)	201 (92.3)	0.040^a^
	Sedentary	21 (10.0)	190 (90.0)	
Contacts of healthy birds with saliva of suspected ones	Nomadic	17 (8.1)	194 (91.9)	0.001^a^
	Sedentary	45 (21.3)	166 (78.7)	
Contacts of healthy birds with fomites contaminated with materials from infected birds	Nomadic	12 (5.7)	199 (94.3)	<0.001^a^
	Sedentary	68 (32.2)	143 (67.8)	

a Statistically significant at *P* < 0.05.

### Pastoralists’ knowledge about transmission of HPAI H5N1 virus among birds

The majority of sedentary (74.4%, 157/211) and few of nomadic (15.6%, 33/211) pastoralists mentioned that contacts of healthy birds with blood of infected ones can be a route for transmission of HPAI H5N1 virus in the flocks: this difference was significant (*P* < 0.05). Less than one‐third of each group significantly mentioned contacts of healthy birds with infected faeces, nasal discharges and saliva to be routes for transmission of HPAI H5N1 virus among birds. Further, about one‐third of sedentary (32.2%, 68/211) and very few of the nomadic (5.7%, 12/211) pastoralists (significant difference at *P* < 0.05) reported that contacts of healthy birds with fomites contaminated with materials from infected birds can serve as a route for transmission for avian influenza virus in poultry flocks (Table 1).

### Identification of risk factors for HPAI H5N1 in pastoral poultry flocks

Less than half of the nomadic (46.9%, 99/211) but more than half of sedentary (60.7%, 128/211) pastoralists (significant at *P* < 0.05) mentioned that birds scavenging around nearby (i.e. <0.1 km) water bodies can be predisposed to HPAI H5N1 virus infection. Also, 38.4% of the nomadic and 18.5% of sedentary pastoralists (significant at *P* < 0.05) reported that wild birds around pastoral settlements can harbour the HPAI H5N1 virus and consequently serve as vehicles for the virus to scavenging poultry. More than two‐thirds of the nomadic (79.1%) and less than half of sedentary (43.1%) pastoralists mentioned that disposal of dead birds in open spaces can be risk factor for spread of HPAI H5N1 virus to scavenging birds. Only a few nomadic (8.1%, 17/211) and sedentary (25.6%, 54/211) pastoralists identified the presence of rodents and stray dogs in the pastoral environment to be risk factors for H5N1 virus in scavenging pastoral birds. However, only 32.7% of the nomadic and 32.7% of sedentary pastoralists significantly reported that keeping of ducks in same flock with other birds can be a risk factor for transmission of HPAI H5N1 virus to pastoral birds (Table 2).

**Table 2:** Pastoralists’ identification of the predisposing risk factors for HPAI H5N1 in pastoral poultry flocks of North‐central Nigeria: 2015–2016

Factor	Pastoralists	Yes *n* (row %)	No *n* (row %)	*P*‐value
Nearby (e.g. <0.1 km) water bodies	Nomadic	99 (46.9)	112 (53.1)	0.004^a^
	Sedentary	128 (60.7)	83 (39.3)	
Wild birds around the pastoral settlements	Nomadic	81 (38.4)	130 (61.6)	0.001^a^
	Sedentary	39 (18.5)	172 (81.5)	
Mixed keeping of different bird species in a flock	Nomadic	56 (26.5)	155 (73.5)	0.001^a^
	Sedentary	105 (49.8)	106 (50.2)	
Disposal of dead birds in open spaces	Nomadic	167 (79.1)	44 (20.9)	<0.001^a^
	Sedentary	91 (43.1)	120 (56.9)	
Introduction of sick bird into the flock	Nomadic	58 (27.5)	153 (72.5)	0.001^a^
	Sedentary	92 (43.6)	119 (56.4)	
Presence of stray dogs and rodents in the environment	Nomadic	17 (8.1)	194 (91.9)	0.001^a^
	Sedentary	54 (25.6)	157 (74.4)	
Keeping of ducks in same flock with other birds	Nomadic	69 (32.7)	142 (67.3)	0.001^a^
	Sedentary	33 (15.6)	178 (84.4)	

a Statistically significant at *P* < 0.05.

### Biosecurity measures practiced against HPAI H5N1 virus infection

Biosecurity measures of disinfecting equipment and premises, and use of personal protective equipment (PPE, such as the skin apron and gloves, etc. for protection during disease outbreaks in their herds) are very important in the control/prevention of avian influenza virus, but were not significantly (*P* > 0.05) mentioned by the pastoralists to be used as mitigation measures against avian influenza (H5N1) in poultry flocks. Majority of the nomadic (76.3%, 161/211) and sedentary (93.8%, 199/211) pastoralists mentioned isolation of sick birds from healthy ones to be a significant biosecurity measure against the disease in their poultry flocks. Also, more than two‐third of the nomadic (82.0%, 173/211) and sedentary (91.9%, 194/211) pastoralists significantly reported that routine cleaning of equipment and premises was a biosecurity measure against HPAI H5N1 virus infection. However, very few nomadic (10.9%) and sedentary (26.1%) pastoralists significantly reported movement restriction of birds to nearby water bodies to be mitigation measures, while 7.6% of the nomadic and 16.1% of sedentary pastoralists mentioned that keeping of birds according to species was a mitigation measure against the disease. Less than one‐third of the nomadic (13.3%) and sedentary (25.6%) pastoralists significantly mentioned burning of dead birds to be biosecurity measures against the disease. Further, low proportions of the nomadic (12.3%, 26/211) and sedentary (31.8%, 67/211) pastoralists significantly reported the practice of dead birds burial to be biosecurity measures against avian influenza in pastoral bird flocks (Table 3).

**Table 3:** Biosecurity measures practised by pastoralists against likelihood of HPAI H5N1 in pastoral poultry flocks of North‐central Nigeria: 2015–2016

Practice	Pastoralists	Yes *n* (row %)	No *n* (row %)	*P*‐value
Isolation of sick birds from flock	Nomadic	161 (76.3)	50 (23.7)	0.001^a^
	Sedentary	198 (93.8)	13 (6.2)	
Cleaning of equipment and premises	Nomadic	173 (82.0)	38 (18.0)	0.002^a^
	Sedentary	194 (91.9)	17 (8.1)	
Disinfection of equipment and premises	Nomadic	6 (2.8)	205 (97.2)	0.216
	Sedentary	11 (5.2)	200 (94.8)	
Movement restriction of birds to nearby water bodies	Nomadic	23 (10.9)	188 (89.1)	0.001^a^
	Sedentary	55 (26.1)	156 (73.9)	
Keeping of birds according to their species	Nomadic	16 (7.6)	195 (92.4)	0.006^a^
	Sedentary	34 (16.1)	177 (83.9)	
Use of personal protective equipment (e.g. skin apron, gloves, etc.)	Nomadic	7 (3.3)	204 (96.7)	0.079
	Sedentary	15 (7.1)	196 (92.9)	
Burial of dead birds	Nomadic	26 (12.3)	185 (87.7)	0.001^a^
	Sedentary	67 (31.8)	144 (68.2)	
Burning of dead birds	Nomadic	28 (13.3)	183 (86.7)	0.001^a^
	Sedentary	54 (25.6)	157 (74.4)	

a Statistically significant at *P* < 0.05.

### Pastoralists’ socio‐demographic characteristics that influenced their overall knowledge about HPAI H5N1

All the socio‐demographic characteristics of the pastoralists, except marital status influenced their overall knowledge about HPAI H5N1 significantly (*P* < 0.05) at univariate analysis. On multivariable logistic regressions, pastoralists in age group 60–69 years were more likely to possess significant knowledge about HPAI H5N1 than those in age group 30–39 years (OR: 3.90, 95% CI: 1.89, 8.03). However, female pastoralists were two times more likely to possess significant knowledge about avian influenza than the males (OR: 1.66; 95% CI: 1.10, 2.53). Also, sedentary pastoralists were two times more likely to have significant knowledge about H5N1 in pastoral birds than the nomadic pastoralists (OR: 1.76; 94% CI: 1.19, 2.61). Furthermore, pastoralists with tertiary education were six times more likely to possess significant knowledge about the disease than those without formal education (OR: 6.04; 95% CI: 2.57, 14.19; Table 4).

**Table 4:** Pastoralists’ socio‐demographic characteristics associated with their overall knowledge about HPAI H5N1 in pastoral poultry flocks of North‐central Nigeria: 2015–2016

Characteristics	Poor knowledge *n* (row %)	Satisfactory knowledge *n* (row %)	Odds ratio	95% Confidence interval	*P*‐value
Age					
30–39	31 (63.3)	18 (36.7)	1.00		
40–49	44 (55.7)	35 (44.3)	1.37	0.65, 3.05	0.406
50–59	69 (45.4)	83 (54.6)	2.07	1.06, 4.01	0.030^a^
60–69	30 (30.6)	68 (69.4)	3.90	1.89, 8.03	0.001^a^
70–79	16 (36.4)	28 (63.6)	3.01	1.29, 7.01	0.010^a^
Gender					
Males	65 (49.6)	66 (50.4)	1.00		
Females	108 (37.1)	183 (62.9)	1.66	1.10, 2.53	0.010^a^
Occupation					
Nomadic	102 (48.3)	109 (51.7)	1.00		
Sedentary	73 (34.6)	138 (65.4)	1.76	1.19, 2.61	0.004^a^
Formal education					
None	191 (69.7)	83 (30.3)	1.00		
Primary	37 (54.4)	31 (45.6)	1.92	1.21, 3.31	0.010^a^
Secondary	25 (49.0)	28 (51.0)	2.57	1.41, 4.68	0.002^a^
Tertiary	8 (27.6)	21 (72.4)	6.04	2.57, 14.19	0.001^a^

a Statistically significant at *P* < 0.05.

### Pastoralists’ socio‐demographic characteristics that influenced their overall practised biosecurity measures against HPAI H5N1

During univariate analysis, all socio‐demographic characteristics significantly influenced the overall practice of biosecurity measures against HPAI H5N1 in pastoral poultry flocks except marital status (*P* < 0.05). On multivariable logistic regressions, only pastoralists in age group 70–79 years were more likely to practise significant biosecurity measures against avian influenza than those in age group 30–39 years (OR: 3.28, 95% CI: 1.99, 7.77). However, female pastoralists were two times more likely to practise significant biosecurity measures against the disease than the males (OR: 1.99; 95% CI: 1.28, 3.09). Also, sedentary pastoralists were two times more likely (OR: 1.88; 94% CI: 1.27, 2.77) to practice significant biosecurity measures against HPAI H5N1 than the nomadic pastoralists. Only pastoralists with tertiary education were more likely to practise significant biosecurity measures against HPAI H5N1 than those without formal education (OR: 2.89; 95% CI: 1.31, 6.37; Table 5).

**Table 5:** Pastoralists’ socio‐demographic characteristics associated with their overall practised biosecurity measures against HPAI H5N1 in pastoral poultry flocks of North‐central Nigeria: 2015–2016

Characteristics	Poor practice *n* (row %)	Satisfactory practice *n* (row %)	Odds ratio	95% Confidence interval	*P*‐value
Age					
30–39	35 (71.4)	14 (28.6)	1.00		
40–49	47 (59.7)	32 (40.3)	1.70	0.79, 3.65	0.177
50–59	88 (57.9)	64 (42.1)	1.81	0.90, 3.65	0.090
60–69	59 (60.2)	39 (39.8)	1.65	0.78, 3.46	0.187
70–79	19 (43.2)	25 (56.8)	3.28	1.99, 7.77	0.006^a^
Gender					
Males	91 (69.5)	40 (30.5)	1.99		
Females	155 (53.3)	136 (46.7)	1.99	1.28, 3.09	0.001^a^
Occupation					
Nomadic	127 (60.2)	84 (39.8)	1.00		
Sedentary	94 (44.5)	117 (55.95)	1.88	1.27, 2.77	0.001^a^
Formal education					
None	175 (63.9)	99 (36.1)	1.00		
Primary	39 (57.4)	29 (42.6)	1.31	0.76, 2.25	0.324
Secondary	31 (60.9)	20 (39.2)	1.14	0.61, 2.10	0.673
Tertiary	11 (37.9)	18 (62.1)	2.89	1.31, 6.37	0.008^a^

a Statistically significant at *P* < 0.05.

## Discussion

There are published reports on HPAI H5N1 in commercial and village chickens (Thomas *et al*. 2005; Wakawa *et al*. 2012; Musa *et al*. 2013; Alders *et al*. 2014), but there is paucity of such information on avian influenza in pastoral bird flocks, which often scavenge and are potentially exposed to avian influenza virus infections. To the best of our knowledge, this study was the first to investigate knowledge/awareness of and biosecurity measures for HPAI H5N1 in pastoral poultry flocks. However, the investigation was crucial as it might influence control and prevention of avian influenza among birds in such potentially exposed communities in Nigeria. The study observed overall significantly poor knowledge and inadequate biosecurity measures practised towards the disease among pastoralists.

This survey found that sources of information about HPAI H5N1 for the pastoralists were from veterinary health officials, relatives and radio broadcasts. This is consistent with a study on sources of information for Chinese communities regarding avian influenza, in which most information was obtained from family and friends rather than from other formal sources (Voeten *et al*. 2009). The different sources of information observed in the survey highlighted a need to provide continuous multiple communication channels on avian influenza to the pastoralists in order to improve their knowledge about the disease. We observed poor knowledge among pastoralists concerning clinical signs indicative of HPAI H5N1 but found high proportions of sedentary pastoralists indicating that the disease could be associated with high morbidity and mortality in susceptible birds. The observed high proportions of sedentary respondents with awareness about the potential high impact of HPAI H5N1 may be due to greater access to information sources compared to the nomadic, and perhaps outbreaks of the disease in some farms around their settlements in the past, since most of the commercial poultry farms are established outside towns and cities in Nigeria. Previous studies reported that HPAI H5N1 causes high morbidity and mortality rates that may approach 90–100% (Swayne 2008; Centers for Disease Control and Prevention (CDC) 2014). This survey found variable proportions of the pastoralists that knew that routes for transmission of H5N1 virus among birds could be through contacts with blood, faeces, nasal secretion and saliva from infected birds as well as fomites on contaminated surfaces in agreement with previous reports (van der Goot *et al*. 2003; CDC 2015, 2016).

Previous studies have indicated that wild birds are reservoir for HPAI H5N1 viruses worldwide (Deogu *et al*. 2003; Hoye *et al*. 2010). Studies have also confirmed the potential risk of backyard flocks roaming in or near water bodies being exposed to interactions with avian Influenza virus‐infected wild birds (Jansen *et al*. 2009; Zheng *et al*. 2010; Desvaux *et al*. 2011). The presence of ponds has been identified as increasing the risk of HPAI outbreaks in the village poultry flocks (Fasina *et al*. 2011; Paul *et al*. 2011). We observed that pastoralists mentioned that potential risk factors for HPAI H5N1 in their poultry flocks included the presence of nearby water bodies (such as ponds) and presence of wild birds around the pastoral settlements. Generally, pastoralists in Nigeria settle in remote areas with scarce human populations and readily available water bodies for their livestock. Such environments can favour birds that may be infected with HPAI viruses and consequently transmit them to scavenging poultry. Pastoralists also reported high contact rates between their birds and wild birds during scavenging.

Previous surveys have reported that mixing of different bird species together increases HPAI H5N1 virus transmission in the flocks (Henning *et al*. 2009; Alhaji & Odetokun 2011; Loth *et al*. 2011). Also, studies have reported presence of ducks in poultry flocks to play potential role as reservoir of HPAI virus (Gilbert *et al*. 2006; Biswas *et al*. 2009; Henning *et al*. 2010; Paul *et al*. 2011); and infected dead birds may be fed upon by rodents, cats and dogs thereby making them reservoirs of HPAI viruses (Food and Agricultural Organization of the United Nations (FAO) 2008). In this survey, pastoralists further indicated that keeping of different poultry species in the same flock, the presence of rodents and stray dogs in their settlements as well as the keeping of ducks among poultry flocks are risk factors for avian influenza infection of pastoral poultry flocks.

Our study found pastoralists mentioned isolation of sick birds from flocks, and cleaning of equipment and premises as biosecurity measures against HPAI H5N1. Cleaning and disinfecting the equipment and premises have been reported to be the most required biosecurity practices because both are particularly effective in interrupting potential HPAI H5N1 viruses (Paul *et al*. 2011), though sustainable use of disinfectants is unlikely in pastoral poultry production system because of the extensive management system (Food and Agricultural Organization of the United Nations (FAO) 2008). Biosecurity practices by the keepers of free‐range flocks cannot act alone; community‐led initiatives are therefore needed (Food and Agricultural Organization of the United Nations (FAO) 2008). Also, in pastoral scavenging bird flocks, the risk mitigation measures (such as birds’ movement restriction) may not outweigh risk propagation practices (scavenging) with consequent frequent introduction of low pathogenic avian influenza (LPAI) viruses. However, this interface maintains the immunity of the birds, preventing infection and reducing the opportunity for viral mutation and development of HPAI as previously reported (Alhaji & Odetokun 2011).

This study found significantly more knowledge about HPAI H5N1 in female pastoralists than in male counterparts. This may be due to domestic cultural economic independence between the genders in pastoralists that create more burden on females, and which encourages them to keep local poultry. Conversely, results from a study in pastoral communities of Ethiopia found an association between sex and knowledge about bovine tuberculosis to be greater in male pastoralists than the females (Gele *et al*. 2009). We found more significant knowledge about HPAI H5N1 in sedentary pastoralists than in the nomadic. Previous studies in similar settings of nomadic and agro‐pastoral communities in Tanzania found that agro‐pastoralists possessed higher predictive overall biomedical knowledge of pulmonary tuberculosis than the nomadics, due to changes of lifestyle, brought about by sedentarization, that improve access to health and social services as well as veterinary extension services (Gele *et al*. 2009; Mengistu *et al*. 2010). This survey observed that significantly only participants in age group 70–79 years practised biosecurity measures against HPAI H5N1. This may be due to their higher understanding of HPAI basic epidemiology acquired from experience. Only pastoralists with tertiary education significantly practised biosecurity measures. To improve biosecurity practices against avian influenza by pastoralists, health education targeting specific socio‐demographic groups is crucial.

As a limitation, this study used data obtained from a cross‐sectional study, which does not show causal relationships, but does demonstrate associations of socio‐demographic variables with knowledge and practised biosecurity measures towards the disease in the pastoral communities. Furthermore, the selection of participants was based on systematic sampling that may have brought in errors and biases. But we believe that this was controlled for in the selection process since pastoral households were homogenous in nature across the study area.

## Conclusion

This study collected preliminary information on knowledge/awareness, risks identification and biosecurity measures on HPAI H5N1. This formed part of the process of reaching communities potentially at risk of the disease. Most pastoralists neither possessed adequate knowledge about avian influenza nor applied adequate biosecurity measures against it, which are the challenging critical gaps. These findings may support preventative education on transboundary diseases among vulnerable poultry flocks in remote areas of developing countries. Education of pastoralists on HPAI virus infection, specifically on information regarding clinical signs of the disease in birds, transmission dynamics among species of birds, flyways of migrating wild birds and adequate mitigation measures are recommended.

## Source of funding

This study was sponsored by the authors themselves without any external financial support.

## Conflicts of interest

The authors declare that they have no conflicts of interest.

## Ethics statement

The authors confirm that the ethical policies of the journal, as noted on the journal's author guidelines page, have been adhered to and the appropriate ethical review committee approval has been received.

## Contributions

The authors were involved in the concept and design of the study, in the collection, analysis and interpretation of data. Both authors contributed critically to revising the manuscript and read and approved the final version.
